# Co-evolution of strain design methods based on flux balance and elementary mode analysis

**DOI:** 10.1016/j.meteno.2015.04.001

**Published:** 2015-05-21

**Authors:** Daniel Machado, Markus J. Herrgård

**Affiliations:** a Centre of Biological Engineering, University of Minho, 4710-057 Braga, Portugal; b The Novo Nordisk Foundation Center for Biosustainability, Technical University of Denmark, 2970 Hørsholm, Denmark

**Keywords:** Metabolic engineering, Rational strain design, Computational methods, Constraint-based modeling

## Abstract

More than a decade ago, the first genome-scale metabolic models for two of the most relevant microbes for biotechnology applications, *Escherichia coli* and *Saccaromyces cerevisiae*, were published. Shortly after followed the publication of OptKnock, the first strain design method using bilevel optimization to couple cellular growth with the production of a target product. This initiated the development of a family of strain design methods based on the concept of flux balance analysis. Another family of strain design methods, based on the concept of elementary mode analysis, has also been growing. Although the computation of elementary modes is hindered by computational complexity, recent breakthroughs have allowed applying elementary mode analysis at the genome scale. Here we review and compare strain design methods and look back at the last 10 years of *in silico* strain design with constraint-based models. We highlight some features of the different approaches and discuss the utilization of these methods in successful *in vivo* metabolic engineering applications.

## Highlights

• Computational strain design methods are divided into two main families.
• We trace the evolutionary history of these two families.
• Surveyed successful cases of model-guided strain design for industrial applications.
• Most proposed methods have not yet been tested in real applications.
• Agreement between in silico and in vivo results shows potential of tested methods.

## 1 Introduction

Computational modeling has emerged as a fundamental tool for unraveling the complexity of biological processes. There are currently many different mathematical formalisms that can be used to model biochemical reaction networks (Machado et al., 2011). Among these formalisms, the constraint-based modeling approach has become widely adopted for large-scale modeling of metabolism (Bordbar et al., 2014). Constraint-based models have been used for a multitude of applications from guiding biological discovery to the improvement of industrial bioprocesses (McCloskey et al., 2013).

Constraint-based models can be used to simulate the cellular phenotype at steady-state using different methods. The most common approach, flux balance analysis (FBA), is a linear programming formulation that relies on the maximization of a cellular objective, such as growth or ATP generation, to determine the steady-state flux distribution through a metabolic network (Orth et al., 2010). Other methods, typically used for simulation of mutant strains, are based on principles of minimization of metabolic and regulatory adjustments (MOMA, ROOM) (Segrè et al., 2002, Shlomi et al., 2005). These kinds of methods are usually classified as biased, since they rely on the assumption of some evolutionary optimization principle to determine a biologically meaningful and physicochemically valid steady-state flux distribution.

There are also unbiased approaches to analyze feasible flux distributions in large-scale metabolic networks, including Monte Carlo sampling and metabolic pathway analysis (Lewis et al., 2012). Elementary mode analysis (EMA) is one of the most popular approaches for metabolic pathway analysis. It provides an unbiased description of the metabolic solution space in terms of minimal sets of reactions that operate in steady-state (Schuster and Hilgetag, 1994). These so-called elementary (flux) modes (EMs) are the basis for several methods to analyze the properties of metabolic networks, including robustness and fragility, as well as to calculate the theoretical yields of all metabolic routes (Trinh et al., 2009).

**Fig. 1:**
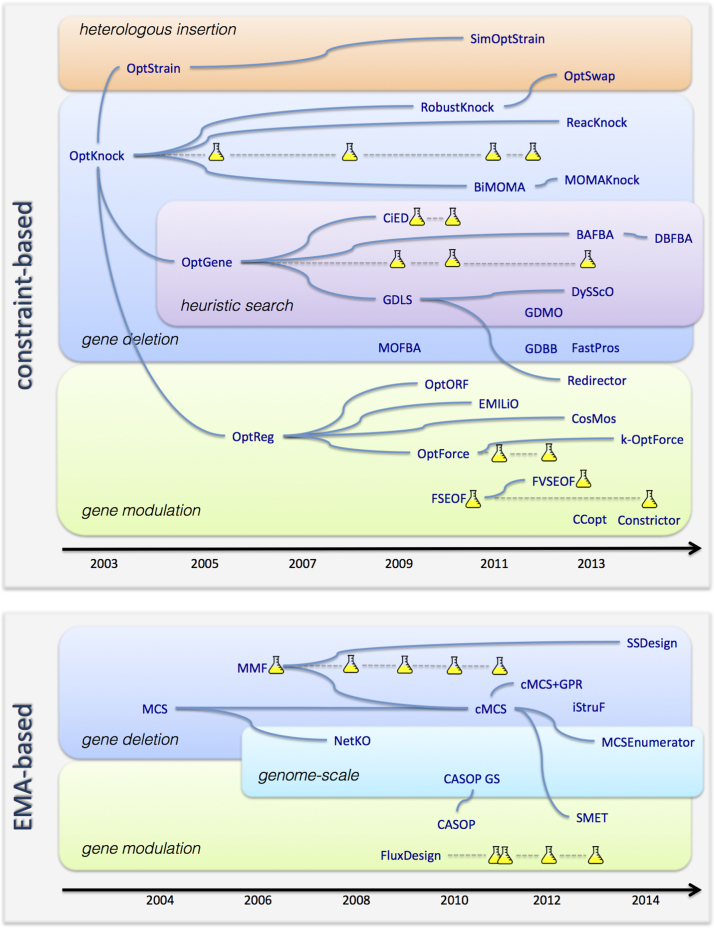
Chronological perspective of the evolution of strain design methods using constraint-based analysis and elementary mode analysis (EMA). Connections represent common features between methods, not necessarily a direct extension of the previous method. The shake flask symbol represents experimental applications of the respective methods.

Both biased and unbiased methods have been used for strain design since the first genome-scale metabolic models of two industrially relevant microbes, *Escherichia coli* and *Saccaromyces cerevisiae*, were published in the early 2000s (Edwards and Palsson, 2000, Förster et al., 2003). From a metabolic engineering perspective, such models can be used for computer-aided design of optimal genetic and culture condition manipulation strategies to improve the production of industrially relevant compounds. However, given the size of metabolic networks, the exhaustive analysis of multiple simultaneous genetic manipulations becomes computationally infeasible. In order to address this challenge, a variety of methodological solutions have been proposed (Fig. 1).

## 2 Constraint-based methods

**Fig. 2:**
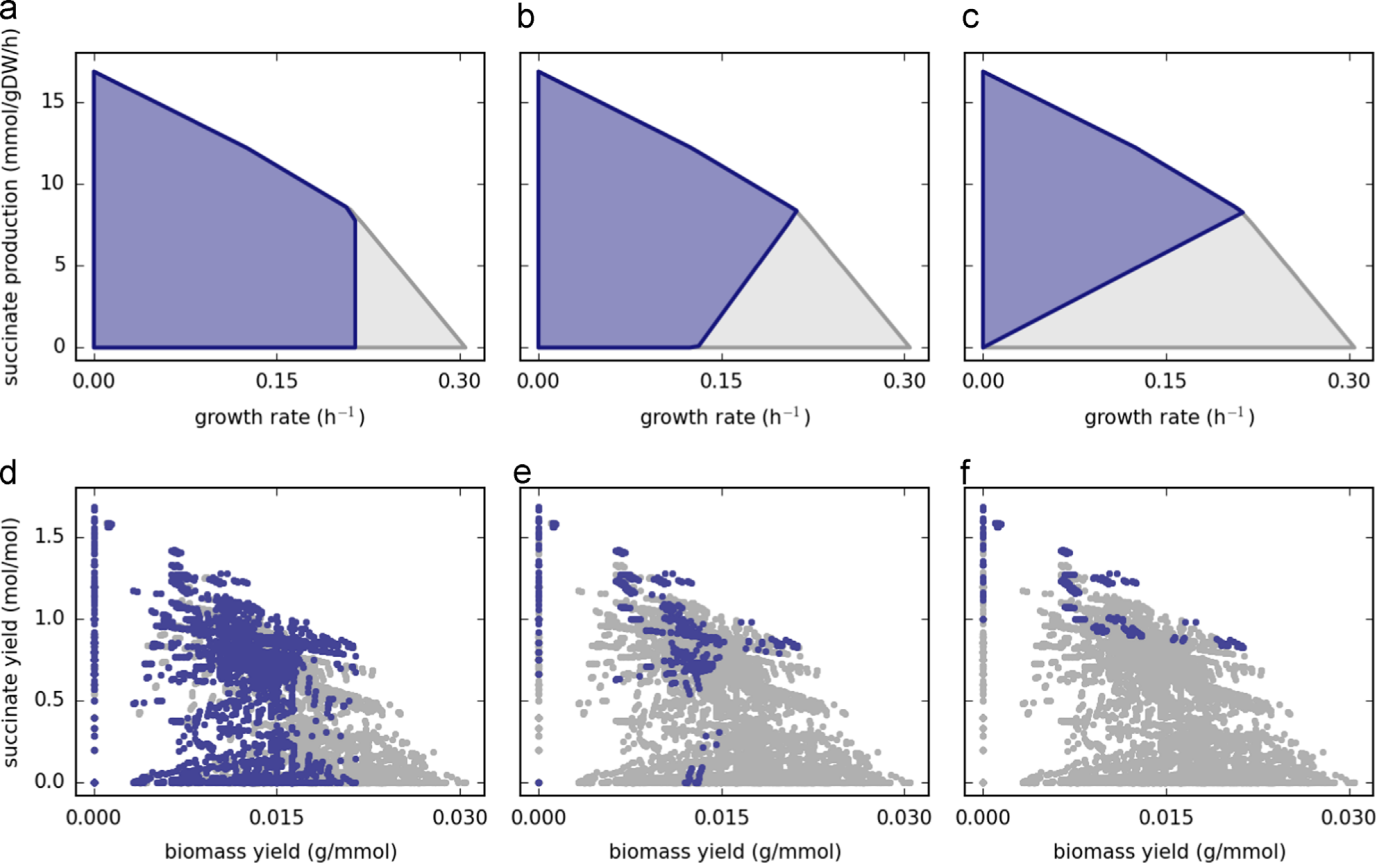
Production envelopes for anaerobic succinate production from glucose based on the *E. coli* core model (Orth et al., 2009) for a maximum glucose uptake rate of 10 mmol/gDW/h (a–c) and the respective EM yield distribution for each solution space (d–f): (a) wild-type strain (light gray) vs. triple-deletion mutant (ACKr, ATPS4r, FUM) resulting in a design without growth-coupling (purple); (b) wild-type strain vs. triple-deletion mutant (ACALD, PYK, ME2) resulting in a partially growth-coupled design; (c) wild-type strain vs. double-deletion mutant (ACALD, LDH_D) resulting in a fully growth-coupled design; (d–f) EM distribution of the wild-type (light gray) and the respective mutant strains in (a–c) (purple). The EM yield locations correspond to vertices in the flux solution space at the maximal glucose uptake rate. (For interpretation of the references to color in this figure caption, the reader is referred to the web version of this paper.)

The first systematic optimization-based method for strain design was the OptKnock approach introduced by Burgard et al. (2003). OptKnock is a bilevel optimization approach that determines reaction deletion strategies to couple the production of a desired compound with cellular growth (Burgard et al., 2003). In OptKnock, the outer optimization layer maximizes the product yield, while the inner layer optimizes for the cellular growth. Using duality theory, this bilevel optimization problem can be reformulated as a single mixed integer linear programming (MILP) problem. Growth-coupled designs represent mutant strains that are forced to carry flux through the target pathway as a requirement to achieve optimal growth rates. When such strains are subject to adaptive evolution they gradually evolve towards the optimal phenotype (Fong et al., 2005). Growth-coupled designs can be visualized by projecting the flux solution space onto the growth and production axes, forming the so-called production envelope (Fig. 2). Two different kinds of growth-coupled designs can be distinguished, which will herein be referred to as *partial* and *full* growth-coupling. In the first case, the strain is only forced to produce the target product at optimal growth rates (Fig. 2b), whereas in the latter case the strain is unable to grow without product formation (Fig. 2c). One limitation of OptKnock is the degeneracy in the solution of the inner problem, which can sometimes result in overly optimistic predictions and lead to strain designs that are not effectively growth-coupled (Fig. 2a).

The introduction of OptKnock has laid the foundation for a diversity of bilevel methods for rational strain design that have been improving over the years. One of such extensions, RobustKnock, uses a max–min strategy to account for the degeneracy in FBA solutions, leading to strain designs that are effectively growth-coupled (Tepper and Shlomi, 2010). An alternative, simpler to implement, approach is to apply objective tilting in the OptKnock formulation (Feist et al., 2010). ReacKnock is a reformulation of OptKnock that differs in the transformation applied to convert the bilevel problem to a single level MILP. A comparison of the two methods shows that higher product yields and faster computations can be obtained with ReacKnock (Xu et al., 2013b). The BiMOMA and MOMAKnock approaches replace the simulation layer in OptKnock with MOMA (Segrè et al., 2002), making it possible to find designs that do not need to couple cellular growth to metabolite production (Kim et al., 2011, Ren et al., 2013).

Although MILP formulations can be used to find a globally optimal solution, their computational cost can, in the worst case, increase exponentially with the number of reaction deletions. A common approach to deal with complex optimization problems is to use heuristic optimization strategies that do not guarantee finding globally optimal solutions, but often find sufficiently good solutions with a reasonable computational cost. Patil and co-workers implemented OptGene, an optimization method based on genetic algorithms, that allows accounting for larger numbers of deletions without an increase in computational cost (Patil et al., 2005). This formulation was later extended to also support simulated annealing as an optimization method (Rocha et al., 2008). Besides increased efficiency for finding reasonably good strain design strategies, these approaches are also more flexible, allowing the implementation of non-linear objective functions. Heuristic optimization methods also allow the utilization of different simulation strategies for the inner problem, such as MOMA and ROOM (Segrè et al., 2002, Shlomi et al., 2005) without the need to significantly change the overall optimization approach.

Other methods using nature-inspired metaheuristics to solve strain design optimization problems include CiED, based on evolutionary algorithms (Fowler et al., 2009), BAFBA, implementing a bees algorithm (Choon et al., 2012), and DBFBA, combining the latter with differential evolution (Choon et al., 2014). Since heuristic methods do not guarantee a globally optimal solution, it is advantageous to experiment with multiple optimization strategies. The hybrid method GDLS combines an heuristic global search approach with a local search method for more efficiently scanning the solution space of genetic designs (Lun et al., 2009).

Gene deletions represent only a subset of the genetic manipulations that can be performed *in vivo* in order to redirect metabolic flux towards a target metabolite. In many applications it is necessary to up-regulate pathways that would become production bottlenecks, or to down-regulate a pathway where a full deletion would otherwise be lethal. OptReg is an extension of OptKnock that accounts for up/down-regulation of gene expression as well as gene deletions (Pharkya and Maranas, 2006). In order to account for the effects of transcriptional regulation, Kim and Reed introduced OptORF, which includes transcriptional regulatory constraints as part of its formulation (Kim and Reed, 2010). With this method it is also possible to simulate manipulations directly at the gene level rather than at the reaction level. Other approaches that account for gene modulation targets include EMILiO (Yang et al., 2011) and CosMos (Cotten and Reed, 2013). These two methods simulate the effect of gene up-regulation by forcing an increase in the flux of the respective reaction, which may not always be realistic as increasing gene or protein expression *in vivo* is not guaranteed to increase the flux through the corresponding reaction. The Redirector method takes a different approach by modeling up-regulations as negative weights in the cellular objective, reflecting the trade-off between cellular growth and the cost of amplifying the desired pathways (Rockwell et al., 2013).

All the methods mentioned so far rely on the simulation of a particular flux distribution for the proposed mutant strain. However, these simulations may not realistically reflect the metabolic response to the proposed genetic perturbations. This discrepancy between *in silico* and *in vivo* response can result in misleading design strategies. A more reliable alternative is to directly compare the metabolic fluxes of the wild-type and the desired mutant strain, and observe which fluxes must necessarily change in order to obtain the desired phenotype. This kind of approach has been implemented in FSEOF (later extended to FVSEOF) using an iterative search method that scans the production envelope to detect flux changes (Choi et al., 2010, Park et al., 2012). The OptForce method allows finding optimal intervention sets of minimal size by first calculating flux variability ranges in a manner similar to FSEOF and then using bilevel optimization to find the minimal set (Ranganathan et al., 2010).

The majority of methods described above focus on predicting which manipulations of native genes would result in increased production of a desired metabolite. However, in actual cell factory development project the focus is often on expression of heterologous genes to enable introducing new biosynthetic capabilities to the host organism. OptStrain, one of the first extensions of OptKnock, was developed to account for heterologous gene expression by inserting reactions that are retrieved from an universal metabolic reaction database (Pharkya et al., 2004). SimOptStrain improves OptStrain by performing additions and deletions simultaneously, resulting in novel design solutions that would not be found otherwise (Kim et al., 2011). The OptSwap method presents the option of replacing native oxidoreductase enzymes with heterologous versions with different co-factor specificities (NADH or NADPH), resulting in growth-coupled designs with significantly higher yields (King and Feist, 2013).

There are few other constraint-based approaches worth mentioning, which are not so commonly referenced and do not fall into the categories described above. These include GDBB, implementing a truncated branch and bound technique (Egen and Lun, 2012); CCopt, based on probabilistic flux bounds (Yousofshahi et al., 2013); FastPros, with a new iterative design based on shadow-price information (Ohno et al., 2013); Constrictor, with combinatorial search of down-regulation targets (Erickson et al., 2014); and multi-objective search methods based on Pareto front analysis such as MOFBA (Oh et al., 2009) and GDMO (Costanza et al., 2012). An in-depth analysis of each of these methods is out of the scope of this review.

## 3 EMA-based methods

Methods based on EMA have been evolving in parallel with the constraint-based methods described previously. In general, elementary mode (EM) enumeration is computationally expensive due to the combinatorial explosion in the number of EMs as a function of network size. On the other hand, since EMs fully describe the steady-state solution space, once the EM set is enumerated, the impact of structural changes in the metabolic network regarding specific phenotypes such as a desirable metabolite production can be assessed very efficiently. For instance, the solution space of a deletion mutant is precisely described by the subset of EMs that do not contain the respective reaction(s).

In 2004, Klamt and Gilles introduced the concept of minimal cut sets (MCS). These are the minimal sets of reaction deletions required to fully block an undesired function in a metabolic network (Klamt and Gilles, 2004). MCSs and EMs are dual properties of metabolic networks, and one can be computed from the other. From a metabolic engineering perspective, this approach can be used to block the production of undesired by-products including common overflow metabolites such as acetate, ethanol, or glycerol, in order to route flux towards a more desirable product.

As an alternative to MCSs, Trinh and co-workers developed the method of minimal metabolic functionality (MMF), a greedy search approach for determining reaction deletions that reduce a metabolic network to its most efficient pathways (Trinh et al., 2006). One advantage of MMF is that a prioritized list of interventions is given, sorted by their impact on network flexibility (i.e. the number of pathways that can carry a given flux).

One limitation of the MCS and MMF approaches is that the elimination of undesired functionality from the network can result in the elimination of desired functionality as well. To address this problem, Hädicke and Klamt introduced constrained minimal cut sets (cMCS) (Hädicke and Klamt, 2011). This is a generalization of MCS to account for a set of desired EMs that must be preserved when the undesired EMs are eliminated. The authors show that other strain-design methods such as MMF, OptKnock and RobustKnock can be implemented as particular cases of cMCS. The SSDesign method is a reformulation of MMF that also includes additional constraints for preserving a minimum set of EMs with desired properties during the elimination steps (Toya et al., 2014).

Two other methods to find optimal deletions include an extension of cMCS with gene-protein-reaction (GPR) constraints, which allows a direct analysis of gene (rather than reaction) deletions (Jungreuthmayer and Zanghellini, 2012), and the iStruF method that searches for reaction deletions based on a flux estimation method named *structural fluxes* (Soons et al., 2013). An application of MCS for gene essentiality prediction at the genome-scale (NetKO) was performed by decomposing the network into so-called *pathway fragments* (Imielinski and Belta, 2008).

EMA-based method have also been used to determine gene up/down-regulation targets that redirect metabolic flux towards the target product. One of the first EMA-based methods to implement this kind of interventions was FluxDesign (Melzer et al., 2009). This method computes the correlations between fluxes through each reaction and the target reaction in the set of EMs. Strongly positive or negative correlations suggest, respectively, overexpression or deletion targets. This method can be considered to be analogous to the FSEOF method introduced above.

CASOP, from the same authors of cMCS, is another method that accounts for gene modulation to maximize, not only yield, but also productivity (Hädicke and Klamt, 2010). This method searches for manipulation strategies with an optimal trade-off between product yield and network flexibility. Here, the network flexibility is used as a measure of network capacity (i.e. the total flux carried by a pathway), which is interpreted as an indicator for the specific production rate.

As mentioned earlier, one of the main limitations of EMA is that the number of EMs grows exponentially with the size of the metabolic network. Since this makes full EM enumeration impractical at the genome scale, a more feasible alternative might be to compute a subset of the full EM set by random sampling (Kaleta et al., 2009, Machado et al., 2012). This kind of approach was implemented in CASOP-GS, which extends CASOP to the genome scale (Bohl et al., 2010).

The issue of computational feasibility was also addressed by the MILP approach introduced by de Figueredo et al. that allows computing the *K-shortest* EMs at the genome-scale. Extending this work, von Kamp and Klamt recently introduced MCSEnumerator, a method to compute the smallest MCSs in genome-scale models by exploiting the duality between MCSs and EMs (von Kamp and Klamt, 2014). With this method the authors were able to enumerate an unprecedented number of intervention strategies, including an exhaustive enumeration of up to 7 simultaneous deletions using a genome-scale model of *E. coli*. The design strategies found by MCSEnumerator included strain design strategies that were not previously found with OptKnock (Feist et al., 2010).

## 4 Applications

Strain design methods are usually published with practical case studies proposing specific strain design strategies. Most of the case studies have used *E. coli* as the model organism as the genome-scale metabolic models for this organism have continued to be the most predictive of all models. The most popular target products considered in the case studies were succinate (20 cases), ethanol (12), lactate (9), acetate (5), fumarate (5) and glycerol (5). Unfortunately, one of the main drawbacks of most methods is the lack of experimental validation of the proposed strain designs, as most of the designs are only evaluated *in silico* by comparison with previously explored experimental design strategies that had proven to be successful. Only four of the methods reviewed herein (CiED, MMF, FSEOF, FVSEOF) have included experimental validation of the design suggestions in their original publication. In some cases (OptORF, Redirector, CASOP, SSDesign) a comparison of the selected manipulation targets with previously published strain designs is presented. However, this does not allow us to make conclusions about quantitative predictions, since the *in silico* and previously experimentally validated designs are only partially the same. Despite this apparent gap between computational work and experimental validation, there are examples of utilization of these methods in successful applications (Table S1).

OptKnock was used to find growth-coupled designs for lactate production in *E. coli* (Fong et al., 2005). Three proposed knock-out designs were implemented *in vivo* and subjected to adaptive evolution with selection for increased growth rate. It was observed that all the strains evolved in the direction of the predicted phenotype of increased L-lactate production. OptKnock was also used for identifying strain designs that increase the respiratory rate of *G. sulfurreducens* (Izallalen et al., 2008), the production of 1,4-butanediol in *E. coli* (Yim et al., 2011), and the production of 2,3-butanediol in yeast (Ng et al., 2012).

OptGene has been used to optimize the production of multiple products in yeast. Deletion targets for non-growth-coupled production of sesquiterpene (Asadollahi et al., 2009) and vanillin (Brochado et al., 2010) were found using MOMA for simulation of the mutant phenotypes. On the other hand, using FBA for simulation, a growth-coupled design strategy for succinate production was obtained with OptGene. The growth-coupled mutant was further optimized by adaptive laboratory evolution, followed by a second round of metabolic engineering, resulting in a final 30-fold improvement in succinate titer (Otero et al., 2013).

Another evolutionary optimization-based approach, CiED, was used in combination with MOMA to find gene deletion strategies to increase the intracellular pools of malonyl-CoA (Fowler et al., 2009) and NADPH (Chemler et al., 2010) in *E. coli*. These are two important precursors for the production recombinant natural products. The resulting strains showed not only an increase in intracellular concentrations of these precursors, but also increased production titers of multiple flavonoids (naringenin, eriodictyol, leucocyanidin, and (+)-catechin).

OptForce was also used to address the increase of intracellular malonyl-CoA concentration for production of heterologous compounds in *E. coli*. A combination of gene overexpression and deletions resulted in a 4-fold increase in malonyl-CoA concentration relative to the wild-type strain (Xu et al., 2011). This method was also used for fatty acid production in *E. coli*, in a study where 39% of the maximum theoretical yield was reached (Ranganathan et al., 2012).

FSEOF and FVSEOF were respectively used to find gene up-regulation targets for lycopene (Choi et al., 2010) and putrescine (Park et al., 2012) production in *E. coli*. Recently, FSEOF was used to find gene up-regulation targets for actinorhodin production in *S. coelicolor*, resulting in a 52-fold increase in product titers (Kim et al., 2014).

Of the EMA-based approaches, only MMF and FluxDesign seem to have been used in practical applications so far. MMF has been used in multiple applications in *E. coli*. In its original publication, this method was used to reach increased yields of biomass on glucose (Trinh et al., 2006). Later, MMF was used to improve the production of ethanol from a mixture of glucose and xylose (Trinh et al., 2008) and from glycerol (Trinh and Srienc, 2009). In both cases the experimentally obtained yields (0.49 and 0.45 g/g) were remarkably close to the theoretical predictions (0.36–0.51 and 0.50 g/g, respectively). Note that these designs require a high number of deletions, compared to methods such as OptKnock, with a total of 8 and 9 gene deletions, respectively. MMF was also used to obtain designs for increased production of isobutanol (Trinh et al., 2011) and diapolycopendial (Unrean et al., 2010). However, in these cases the match between the predicted and experimental yields was not was not as good as in the case of ethanol production.

FluxDesign was used to find gene up-regulation and deletion targets for the production of lysine in *C. glutamicum* (Neuner and Heinzle, 2011, Becker et al., 2011). In one case a high yield of 0.55 (g/g glucose) was reached (Becker et al., 2011). This method was also used to improve isobutanol production in *B. subtilis* (Li et al., 2012), and poly-hydroxyalkanoates production in *P. putida* (Poblete-Castro et al., 2013). In the latter study, the experimental results confirmed the design suggested by FluxDesign and disproved a different design that had been proposed with OptKnock.

Finally, it is important to highlight the numerous model-guided applications that do not use targeted optimization methods like the ones described so far. We performed a literature survey, expanding a previous survey by Xu et al. (2013a) (Table S1). It can be observed that the production titers and yields of many products have been improved with model-guided simulations without the use of methods for optimized search of genetic modifications. Lycopene titers in *E. coli* were increased up to 8.5-fold using a model-guided strategy that included combinatorial simulations of single, double and triple deletion mutants (Alper et al., 2005a, Alper et al., 2005b). Other applications of combinatorial simulation of gene deletions in *E. coli* have resulted in the development of high producing strains, including a 7-fold increase in succinate titer (Lee et al., 2005), a 7.4-fold increase in molar yield of 3HP production from glycerol (Tokuyama et al., 2014), and a high production yield of L-valine (0.378 g/g) from glucose (Park et al., 2007). Instead of simulating all gene deletions, one can also combine biological intuition on potential manipulation candidates with computational analysis to observe the flux response of the target reaction with respect to the given candidates (flux response analysis). This method was used to select gene manipulations in *E. coli* that achieved a high yield of L-threonine (0.393 g/g) from glucose (Lee et al., 2007). This method has also be used to determine optimal feeding strategies in fed-batch cultivations (Park et al., 2011).

## 5 Discussion

Strain design approaches based on flux balance and elementary mode analysis have been co-evolving independently. In both cases, the genetic designs have been extended to include not only gene deletions but also gene up/down-regulation and addition of heterologous genes.

The number of constraint-based methods that have been suggested for strain design is significantly higher than the number of EMA-based methods. This is a likely consequence of the computational limitations of the latter, requiring access to high performance computing resources. Given the recent advances that have raised the power of EMA to the genome scale (de Figueiredo et al., 2009, von Kamp and Klamt, 2014) it is possible that EMA-based approaches will be increasingly explored. Constraint-based approaches have limitations in their predictive power due to the potential biases introduced by using phenotype simulation to predict metabolic flux distributions. Recent methods have begun to circumvent this problem by comparing flux ranges instead of simulating particular solutions for each genetic modification (Ranganathan et al., 2010, Choi et al., 2010).

With the decreasing bias of constraint-based methods, the increasing ability of EMA-based methods to compute with larger networks, and the support for more types of genetic modifications for both methods, the gap between these approaches is diminishing. However, there are still fundamental differences between the methods that may lead to different results. Most constraint-based methods search for genetic modifications that shape the solution space in a way that favors the coupling between growth and production of the target compound at the point of optimality (i.e. partial growth-coupling) (Fig. 2b). Reaching a suitable shape may require multiple, possibly unintuitive, simultaneous modifications. During *in silico* simulations, the effect of single modifications on product flux is negligible until a suitable combination is found, making it difficult to implement an iterative strain engineering process where only a few genetic modifications at a time are introduced.

On the other hand, EMA-based methods try to eliminate (or decrease the utilization of) EMs that do not produce the target compound. A fully growth-coupled design is obtained if such EMs are completely eliminated (Fig. 2f). However, each modification alters the EM distribution in the solution space even if the production envelope is unaltered. Since the impact of a modification is measured by the number of affected EMs, there are no “silent” modifications in EMA-based design. This allows utilizing an iterative strain engineering process where only a few modifications at a time are introduced. While this is an advantage of EM-based approaches, the resulting designs tend to contain a larger number of modifications compared to constraint-based designs.

The lack of experimental validation hampers a critical comparison of the different methods. The cases of successful applications that have been published so far seem to be concentrated around a few well established methods. The successful results show that strain design methods are indeed a useful tool for guiding metabolic engineering applications. However, it is not straightforward to make a quantitative assessment of the expected results, since the predictions are based on yields and in many cases only the final product titers are reported.

Growth-coupled designs obtained with FBA seem to result in a good match between *in silico* and *in vivo* results after adaptation of the mutant strains (Fong et al., 2005, Otero et al., 2013). This is consistent with the observation that FBA is suitable to predict the phenotype of evolved strains (Lewis et al., 2010). For MOMA-based predictions there can be some discrepancy between predicted and experimental yields (Asadollahi et al., 2009), which is probably true for non-growth-coupled strain designs in general. In these cases, as well as for designs based on gene modulation, the proposed interventions should be regarded as qualitative, rather than quantitative, predictions. Employing these types of predictions *in vivo* will require testing multiple alternative predictions in a combinatorial fashion to find the optimal design.

So far, EMA-based designs have been computed based on core metabolic models. Although these models have a limited scope compared to genome-scale models, this may not reduce their applicability in metabolic engineering. A possible reason is that, in most strain designs, the intervention targets are enzymes in central metabolic pathways. With designs obtained with core models, the system-wide impact of the proposed genetic modifications can be simulated using genome-scale models. For instance, the growth-coupled design shown in Fig. 2c does not result in growth coupling of succinate production in the respective genome-scale model (Feist et al., 2007). This is due to an alternative pathway for acetaldehyde production from acetyl-CoA using 2-amino-3-oxobutanoate and allo-threonine as intermediates. This alternative pathway can be eliminated with an additional knockout of one of the steps. Hence, the designs obtained with core models are often subsets of more complex designs that can be obtained with genome-scale models. Succinate overproducing mutants have been successfully obtained by deletion of the central acetaldehyde dehydrogenase without eliminating the alternative acetaldehyde production pathway (Sánchez et al., 2006, Jantama et al., 2008). Hence, it is unlikely that most of the carbon flux would be rerouted through this pathway in the mutant strain, but if this strain is subjected to adaptive evolution under growth selection, the alternative pathway may get activated thus reducing succinate production. Experimental results show that EMA-based predictions using core models are highly accurate for central carbon metabolism (Trinh et al., 2008, Trinh and Srienc, 2009). However, the predictive power of EMA-based designs seems to decrease when heterologous pathways are considered, indicating limitations in these heterologous pathways themselves as opposed to limitations in central metabolic precursor generation (Unrean et al., 2010, Trinh et al., 2011).

One of the most important problems in bioprocess engineering is the trade-off between yield, titer, and productivity, which are often conflicting objectives. Since constraint-based models can only make yield-based predictions, other parameters can only be targeted indirectly (Patil et al., 2005, Hädicke and Klamt, 2010). The recent DySScO framework addresses this problem by integrating dynamic flux balance analysis with GDLS (Zhuang et al., 2013). Another solution for this problem is the integration of kinetic and constraint-based modeling, in order to calculate absolute flux rates. This kind of integration was recently implemented in k-OptForce (Chowdhury et al., 2014). Similarly, the recent method SMET combines EM analysis with ensemble modeling to identify rate limiting steps (Flowers et al., 2013).

With the algorithmic improvements and the increasing availability of computational power, the evolution of strain design methods will certainly become less driven by computational efficiency, and more importantly so by the biological plausibility of their results. Recent impressive developments in synthetic biology methods for genome engineering such as the *CRISPR/Cas9* system (Sander and Joung, 2014) will allow implementing more complex designs *in vivo*. Systematic evaluations of *in silico* strain design predictions from different methods will hopefully become the norm rather than the exception in the future.

## Appendix A Supplementary data

Application 1
