# Supplementary material for: Co-evolution of strain design methods based on flux balance and elementary mode analysis
Source: Metab Eng Commun. 2015 May 21;2:85–92. doi: 10.1016/j.meteno.2015.04.001 (PMC8193246; doi:10.1016/j.meteno.2015.04.001)
Supplement: Application 1 [file mmc1.pdf]

Supp Table 1. Literature compilation of successful applications of model-guided strain design towards industrial production of several products. Some applications use constraint-based analysis methods not mentioned in the main text: flux response analysis (FRA), flux variability analysis (FVA), and flux sensitivity analysis (FSA).

First Author	Year	Journal	Organism	Target	Method	Results
Agren	2013	J Ind Micro Biotech	*S. cerevisiae*	succinate	FBA	0.02 (C-mol/C-mol) yield
Alper	2005	Met Eng	*E. coli*	lycopene	MOMA	40% increase in titer
Alper	2005	Nat Biotech	*E. coli*	lycopene	MOMA	8.5-fold increase in titer
Asadollahi	2009	Met Eng	*S. cerevisiae*	sesquiterpene	OptGene	85% increase in titer
Becker	2011	Met Eng	*C. glutamicum*	L-Lysine	FluxDesign	0.55 g/g yield, 120g/L titer, 4.0 g/L/h productivity
Boghigian	2012	Appl Microbiol Biotech	*E. coli*	taxadiene	MOMA	12-fold increase in titer
Bro	2006	Met Eng	*S. cerevisiae*	ethanol	FBA	3% increase in yield (g/g)
Brochado	2010	Micro Cell Fact	*S. cerevisiae*	vanillin	OptGene	1.5-fold yield (g/g) increase
Chemler	2010	Met Eng	*E. coli*	NADPH	CiED	817 mg/L leucocyanidin, 39 mg/L (+)-catechin
Choi	2010	Appl Env Microbiol	*E. coli*	lycopene	FSEOF	up to 283 mg/L titer
Fong	2005	Biotech & Bioeng	*E. coli*	lactate	OptKnock	up to 1.75 g/L titer
Fowler	2009	Appl Env Microbiol	*E. coli*	malonyl-CoA	CiED	100 mg/L/OD naringenin, 55 mg/L/OD eriodictyol
Huang	2012	Appl Microbiol Biotech	*S. roseosporus*	daptomycin	FVA	43.2% improve in yield (g/g)
Izallalen	2008	Met Eng	*G. sulfurreducens*	respiratory rate	OptKnock	increased respiratory rate in mutant
Jung	2010	Biotech & Bioeng	*E. coli*	polylactic acid	MOMA, FRA	successful production of multiple polymers
Kennedy	2009	Genetics	*S. cerevisiae*	formate	FBA	16.5-fold increase in titer
Kim	2014	Biotech J	*S. coelicolor*	actinorhodin	FSEOF	52-fold increase in titer
Lee	2005	Appl Env Microbiol	*E. coli*	succinate	FBA	7-fold increase in titer
Lee	2007	Mol Sys Biol	*E. coli*	L-threonine	FBA, FRA	yield 0.393 g/g, titer 82.4 g/L
Li	2012	Micro Cell Fact	*B. subtilis*	isobutanol	FluxDesign	0.36 (C-mol/C-mol) yield, 5.5 g/L titer
Meng	2011	Biotech Bioproc Eng	*E. coli*	taxadiene	FBA	876 mg/L titer, 8.9 mg/L/h productivity
Moon	2008	Biochem Eng J	*E. coli*	malic acid	FBA	9.25 g/L titer
Neuner	2011	Biotech J	*C. glutamicum*	lysine	FluxDesign (adapted)	up to 9% and 15% yield (C-mol/C-mol)
Ng	2012	Micro Cell Fact	*S. cerevisiae*	2,3-butanediol	OptKnock	0.113 (g/g) yield, 2.29 g/L titer
Oddone	2009	Met Eng	*L. lactis*	GFP	DFBA, FSA	improved 15% yield of GFP
Otero	2013	PLoS ONE	*S. Cerevisiae*	succinate	OptGene	43-fold improvement in yield (g/gDW)
Park	2012	BMC Sys Biol	*E. coli*	putrescene	FVSEOF	up to 0.223 (g/g) yield
Park	2007	PNAS	*E. coli*	L-valine	MOMA	0.378 g/g yield
Park	2011	Biotech & Bioeng	*E. coli*	L-valine	FRA	32.3 g/L titer
Poblete-Castro	2013	Met Eng	*P. Putida*	PHAs	FluxDesign	80% increase in yield, 100% increase in titer
Ranganathan	2011	Met Eng	*E. coli*	fatty acids	OptForce	0.14 (g/g) yield, 1,7 g/L titer
Santala	2011	Micro Cell Fact	*A. baylyi*	triacylglycerol	FBA	5.6-fold increase in yield (mg/gDW)
Tokuyama	2014	Micro Cell Fact	*E. coli*	3HP	FBA	7.4-fold increase in yield (C-mol/C-mol)
Trinh	2006	Met Eng	*E. coli*	biomass	MMF	0.57 (gDW/g) yield
Trinh	2008	Appl Env Microbiol	*E. Coli*	ethanol	MMF	39.07 g/L titer
Trinh	2009	Appl Env Microbiol	*E. coli*	ethanol	MMF	0.45 (g/g) yield
Trinh	2011	Appl Env Microbiol	*E. coli*	isobutanol	MMF	1.75 g/L titer
Unrean	2010	Met Eng	*E. coli*	DPL	MMF	0.17 (mg/g) yield
van Ooyen	2012	Biotech & Bioeng	*C. glutamicum*	L-lysine	FBA	0.34 (mol/mol) yield
Wang	2006	App Gen Mol Biotech	*E. coli*	succinate	FBA, MFA	1.29 (mol/mol) yield
Xu	2012	PLoS ONE	*S. Cerevisiae*	fumarate	FBA	1.675 g/L titer
Xu	2011	Met Eng	*E. coli*	malonyl-CoA	OptForce	4-fold increase in mal-coa, 474 mg/L titer of naringenin
Yim	2011	Nat Chem Biol	*E. coli*	1,4-butanediol	OptKnock	18 g/L titer
